# Correction: Whole-genome sequencing analysis reveals new susceptibility loci and structural variants associated with progressive supranuclear palsy

**DOI:** 10.1186/s13024-024-00763-3

**Published:** 2024-10-14

**Authors:** Hui Wang, Timothy S. Chang, Beth A. Dombroski, Po-Liang Cheng, Vishakha Patil, Leopoldo Valiente-Banuet, Kurt Farrell, Catriona Mclean, Laura Molina-Porcel, Alex Rajput, Peter Paul De Deyn, Nathalie Le Bastard, Marla Gearing, Laura Donker Kaat, John C. Van Swieten, Elise Dopper, Bernardino F. Ghetti, Kathy L. Newell, Claire Troakes, Justo G. de Yébenes, Alberto Rábano-Gutierrez, Tina Meller, Wolfgang H. Oertel, Gesine Respondek, Maria Stamelou, Thomas Arzberger, Sigrun Roeber, Ulrich Müller, Franziska Hopfner, Pau Pastor, Alexis Brice, Alexandra Durr, Isabelle Le Ber, Thomas G. Beach, Geidy E. Serrano, Lili-Naz Hazrati, Irene Litvan, Rosa Rademakers, Owen A. Ross, Douglas Galasko, Adam L. Boxer, Bruce L. Miller, Willian W. Seeley, Vivanna M. Van Deerlin, Edward B. Lee, Charles L. White, Huw Morris, Rohan de Silva, John F. Crary, Alison M. Goate, Jeffrey S. Friedman, Yuk Yee Leung, Giovanni Coppola, Adam C. Naj, Li-San Wang, P. S. P. genetics study group, Clifton Dalgard, Dennis W. Dickson, Günter U. Höglinger, Gerard D. Schellenberg, Daniel H. Geschwind, Wan-Ping Lee

**Affiliations:** 1 Department of Pathology and Laboratory Medicine, Perelman School of Medicine, University of Pennsylvania, Philadelphia, PA USA; 2 Penn Neurodegeneration Genomics Center, Perelman School of Medicine, University of Pennsylvania, Philadelphia, PA USA; 3 Movement Disorders Programs, Department of Neurology, David Geffen School of Medicine, University of California, Los Angeles, Los Angeles, CA USA; 4 Department of Pathology, Department of Artificial Intelligence & Human Health, Nash Family, Department of Neuroscience, Ronald M. Loeb Center for Alzheimer’s Disease, Friedman Brain, Institute, Neuropathology Brain Bank & Research CoRE, Icahn School of Medicine at Mount Sinai, New York, NY USA; 5 Victorian Brain Bank, The Florey Institute of Neuroscience and Mental Health, Parkville, VIC Australia; 6 Alzheimer’s Disease and Other Cognitive Disorders Unit. Neurology Service, Hospital Clínic, Fundació Recerca Clínic Barcelona (FRCB). Institut d’Investigacions Biomediques August Pi I Sunyer (IDIBAPS), University of Barcelona, Barcelona, Spain; 7 Neurological Tissue Bank of the Biobanc-Hospital Clínic-IDIBAPS, Barcelona, Spain; 8 Movement Disorders Program, Division of Neurology, University of Saskatchewan, Saskatoon, SK Canada; 9 Laboratory of Neurochemistry and Behavior, Experimental Neurobiology Unit, University of Antwerp, Wilrijk (Antwerp), Belgium; 10 Department of Neurology, University Medical Center Groningen, NL-9713 AV, Groningen, Netherlands; 11 Fujirebio Europe NV, Technologiepark 6, 9052 Ghent, Belgium; 12 Department of Pathology and Laboratory Medicine and Department of Neurology, Emory University School of Medicine, Atlanta, GA USA; 13 Netherlands Brain Bank and Erasmus University, Rotterdam, Netherlands; 14 Department of Pathology and Laboratory Medicine, Indiana University School of Medicine, Indianapolis, IN USA; 15 London Neurodegenerative Diseases Brain Bank, King’s College London, London, UK; 16 Autonomous University of Madrid, Madrid, Spain; 17 Fundación CIEN (Centro de Investigación de Enfermedades Neurológicas) - Centro Alzheimer Fundación Reina Sofía, Madrid, Spain; 18 Department of Neurology, Philipps-Universität, Marburg, Germany; 19 German Center for Neurodegenerative Diseases (DZNE), Marburg, Germany; 20 Parkinson’s Disease and Movement Disorders Department, HYGEIA Hospital, Athens, Greece; 21 European University of Cyprus, Nicosia, Cyprus; 22 Department of Psychiatry and Psychotherapy, University Hospital Munich, Ludwig-Maximilians-University Munich, Munich, Germany; 23 Center for Neuropathology and Prion Research, Ludwig-Maximilians-University Munich, Munich, Germany; 24 Institute of Human Genetics, Justus-Liebig University Giessen, 35392 Giessen, Germany; 25 Unit of Neurodegenerative Diseases, Department of Neurology, University Hospital Germans Trias I Pujol, Badalona, Barcelona, Spain; 26 Neurosciences, The Germans Trias I Pujol Research Institute (IGTP) Badalona, Barcelona, Spain; 27 Sorbonne Université, Paris Brain Institute – Institut du Cerveau – ICM, Inserm U1127, CNRS UMR 7225, APHP - Hôpital Pitié-Salpêtrière, Paris, France; 28 Banner Sun Health Research Institute, Sun City, AZ USA; 29 University McGill, Montreal, QC Canada; 30 Department of Neuroscience, University of California, San Diego, CA USA; 31 VIB Center for Molecular Neurology, University of Antwerp, Antwerp, Belgium; 32 Department of Neuroscience, Mayo Clinic Jacksonville, Jacksonville, FL USA; 33 Memory and Aging Center, University of California, San Francisco, CA USA; 34 Center for Neurodegenerative Disease Research, University of Pennsylvania School of Medicine, Philadelphia, PA USA; 35 University of Texas Southwestern Medical Center, Dallas, TX USA; 36 Departmento of Clinical and Movement Neuroscience, University College of London, London, UK; 37 Reta Lila Weston Institute, UCL Queen Square Institute of Neurology, London, UK; 38 Department of Genetics and Genomic Sciences, New York, NY, USA; Icahn School of Medicine at Mount Sinai, New York, NY USA; 39 Friedman Bioventure, Inc., Del Mar, CA USA; 40 Department of Psychiatry, Semel Institute for Neuroscience and Human Behavior, University of California, Los Angeles, CA USA; 41 Department of Biostatistics, Epidemiology, and Informatics, Perelman School of Medicine, University of Pennsylvania, Philadelphia, PA USA; 42 Department of Anatomy Physiology and Genetics, the American Genome Center, Uniformed Services University of the Health Sciences, Bethesda, MD USA; 43 Department of Neurology, LMU University Hospital, Ludwig-Maximilians-Universität (LMU) München; German Center for Neurodegenerative Diseases (DZNE), Munich, Germany; and Munich Cluster for Systems Neurology (SyNergy), Munich, Germany; 44 Department of Human Genetics, David Geffen School of Medicine, University of California, Los Angeles, Los Angeles, CA USA; 45 Institute of Precision Health, University of California, Los Angeles, Los Angeles, CA USA

**Correction** **:** **Mol Neurodegeneration 19, 61 (2024)**

**https://doi.org/10.1186/s13024-024-00747-3**

The original article [1] erroneously gives a wrong affiliation for Ulrich Müller. His correct affiliation is Institute of Human Genetics, Justus-Liebig University Giessen, 35392 Giessen, Germany.
