# Supplementary material for: Whole-genome sequencing analysis reveals new susceptibility loci and structural variants associated with progressive supranuclear palsy
Source: Mol Neurodegener. 2024 Aug 16;19:61. doi: 10.1186/s13024-024-00747-3 (PMC11330058; doi:10.1186/s13024-024-00747-3)
Supplement: Supplementary file 1 — Supplementary Material 1. [file 13024_2024_747_MOESM1_ESM.docx]

Whole-Genome Sequencing Analysis Reveals New Susceptibility Loci and Structural Variants Associated with Progressive Supranuclear Palsy

Hui Wang^1,2^*, Timothy S Chang^3^*, Beth A Dombroski^1,2^, Po-Liang Cheng^1,2^, Vishakha Patil^3^, Leopoldo Valiente-Banuet^3^, Kurt Farrell^4^, Catriona Mclean^5^, Laura Molina-Porcel^6,7^, Alex Rajput^8^, Peter Paul De Deyn^9,10^, Nathalie Le Bastard^11^, Marla Gearing^12^, Laura Donker Kaat^13^, John C Van Swieten^13^, Elise Dopper^13^, Bernardino F Ghetti^14^, Kathy L Newell^14^, Claire Troakes^15^, Justo G de Yébenes^16^, Alberto Rábano-Gutierrez^17^, Tina Meller^18^, Wolfgang H Oertel^18^, Gesine Respondek^19^, Maria Stamelou^20,21^, Thomas Arzberger^22,23^, Sigrun Roeber^24^, Pau Pastor^25,26^, Alexis Brice^27^, Alexandra Durr^27^, Isabelle Le Ber^27^, Thomas G Beach^28^, Geidy E Serrano^28^, Lili-Naz Hazrati^29^, Irene Litvan^30^, Rosa Rademakers^31,32^, Owen A Ross^32^, Douglas Galasko^30^, Adam L Boxer^33^, Bruce L Miller^33^, Willian W Seeley^33^, Vivanna M Van Deerlin^1^, Charles L White III^34^, Huw Morris^35^, Rohan de Silva^36^, John F Crary^4^, Alison M Goate^37^, Jeffrey S Friedman^38^, Yuk Yee Leung^1,2^, Giovanni Coppola^3,39^, Adam C Naj^1,2,40^, Li-San Wang^1,2^, PSP genetics study group, Dennis W Dickson^32^#, Günter U Höglinger^41^#, Gerard D Schellenberg^1,2^#, Daniel H Geschwind^3,42,43^#, Wan-Ping Lee^1,2^#

*These authors contributed equally to this work.

#These authors are corresponding authors.

Contents

[Supplementary Figures 3](#_Toc156833801)

[Figure S1. Genome-wide association analysis for whole genome sequencing data. 3](#_Toc156833802)

[Figure S2. Genome-wide significant signals in *MAPT*, *MOBP*, and *STX6*. 4](#_Toc156833803)

[Figure S3. Genome-wide significant signals for *APOE E4,* *APOE E2*. 5](#_Toc156833804)

[Figure S4. Visualization of the addictive risk between APOE4/2 and H1/H2 haplotype in PSP. 6](#_Toc156833805)

[Figure S5. Suggestive signals for *SLCO1A2,* *DUSP10*, and *SP1*. 7](#_Toc156833806)

[Figure S6. Suggestive signals for *FCHO1*/*MAP1S* and *KIF13A*. 8](#_Toc156833807)

[Figure S7. Suggestive signals for *TRIM24*, *TNXB*, and *ELOVL1*. 9](#_Toc156833808)

[Figure S8. Functional analysis of *ZNF592* and the C1 module. 10](#_Toc156833809)

[Figure S9. Genome-wide association analysis for structural variants. 11](#_Toc156833810)

[Figure S10. Samplot for genome-wide significant deletions. 12](#_Toc156833811)

[Figure S11. Deletions in immunoglobulin heavy locus (IGH). 13](#_Toc156833812)

[Figure S12. Samplot for common deletions and duplications in the H1/H2 region. 14](#_Toc156833813)

[Figure S13. Samplot for rare deletions and duplications in the H1/H2 region. 15](#_Toc156833814)

[Figure S14. Counts of ALT alleles by sequencing center. 16](#_Toc156833815)

[Supplementary Methods 17](#_Toc156833816)

[Cohorts with seletion bias against APOE ε2/ε4 17](#_Toc156833817)

[APOE genotype validation 17](#_Toc156833818)

[Masked regions in genome 18](#_Toc156833819)

[Aggregated test for rare variants 18](#_Toc156833820)

[References 19](#_Toc156833821)

[Acknowledgements 20](#_Toc156833822)

[ADSP (sa000001) data: 20](#_Toc156833823)

[ADNI (sa000002) data: 22](#_Toc156833824)

[FASe_Families (sa000004) data: 22](#_Toc156833825)

[KnightADRC (sa000008) data: 23](#_Toc156833826)

[AMP-AD (sa000011) data: 23](#_Toc156833827)

[UPitt Kamboh (sa000012) data: 23](#_Toc156833828)

[NACC Genentech (sa000013) data: 23](#_Toc156833829)

[CacheCounty (sa000014) data: 24](#_Toc156833830)

Supplementary Figures


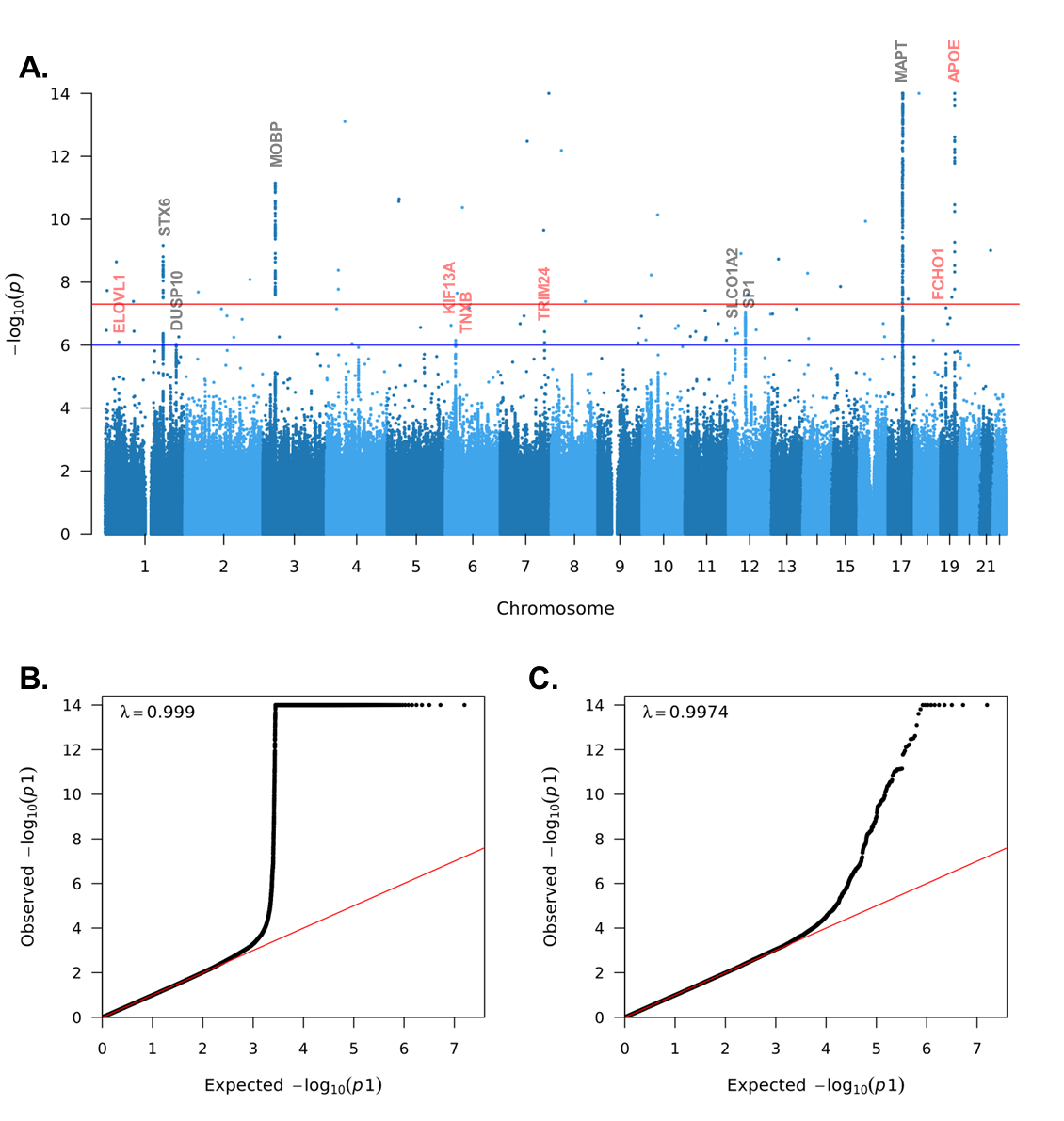


Figure S1. Genome-wide association analysis for whole genome sequencing data.

**A.** Manhattan plot for SNV and INDEL association including variants without supporting variants in LD. Variants with a P value below 1 × 10^-14^ are not shown. Loci with a suggestive or genome-wide significant signal are annotated (new loci in red, known loci in black). The red horizontal line represents genome-wide significance level (5 × 10-8). The blue horizontal line represents suggestive significance level (1 × 10-6). **B.** Q-Q plot for the association analysis. **C.** Q-Q plot for the association analysis after removing the MAPT region.


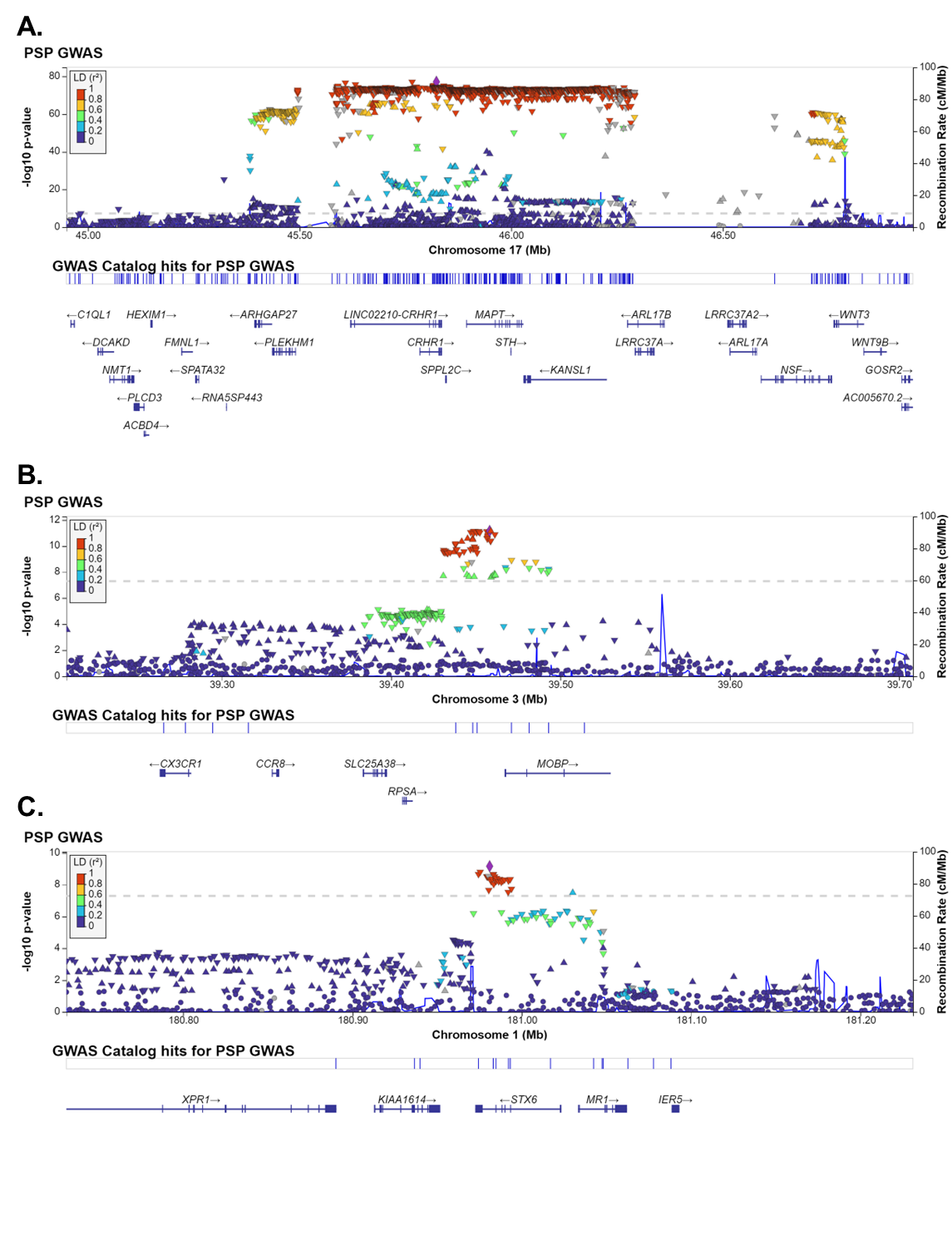


Figure S2. Genome-wide significant signals in *MAPT*, *MOBP*, and *STX6*.

**A.** Significant signals in *MAPT*. **B.** Significant signals in *MOBP*. **C.** Significant signals in *STX6*.


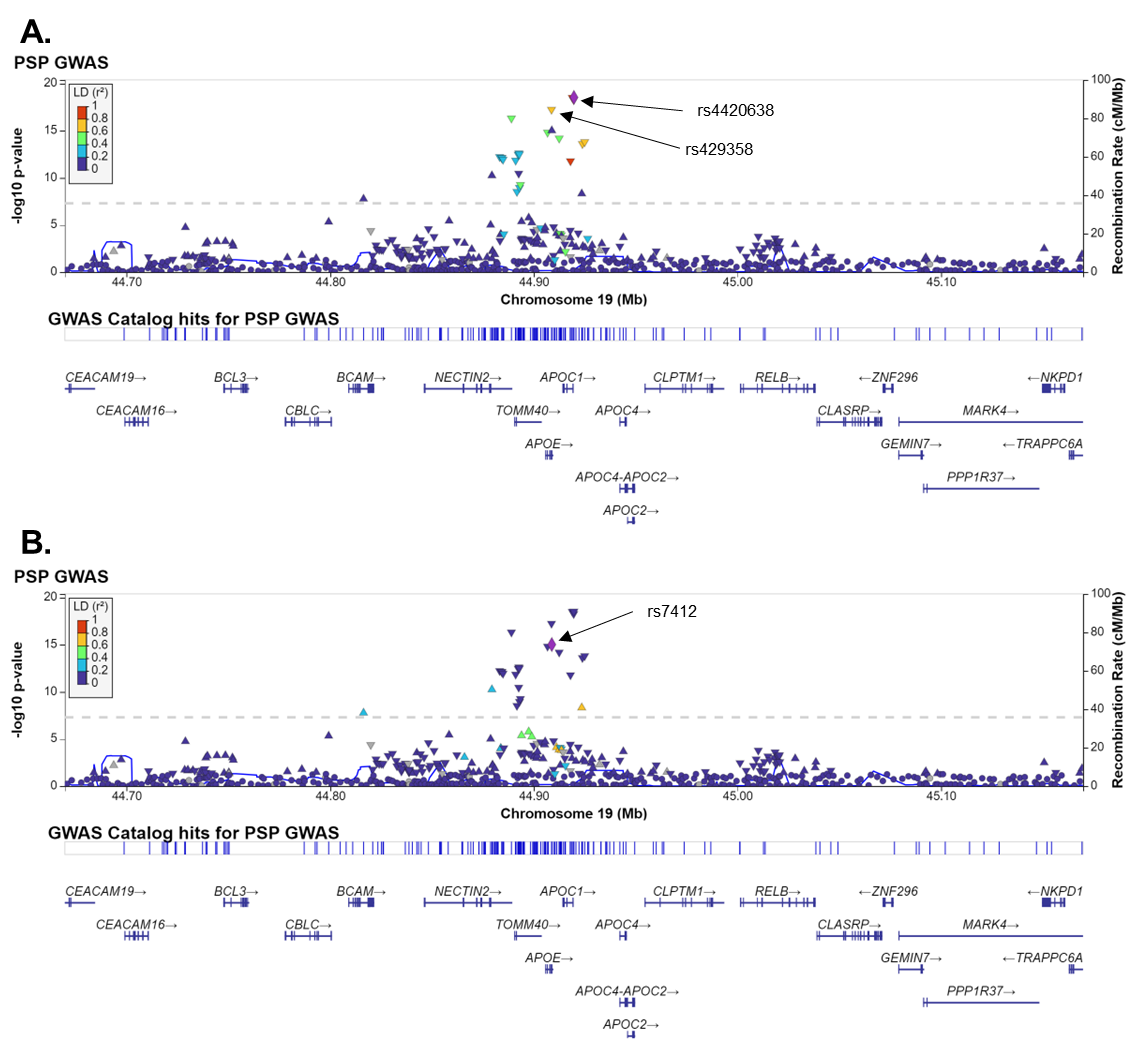


Figure S3. Genome-wide significant signals for *APOE E4,* *APOE E2*.

**A.** Significant signals in *APOE* (Using rs4420638 as linkage disequilibrium (LD) reference). **B.** Significant signals in *APOE* (Using rs7412 as LD reference). LD is calculated on 1000G Europeans.


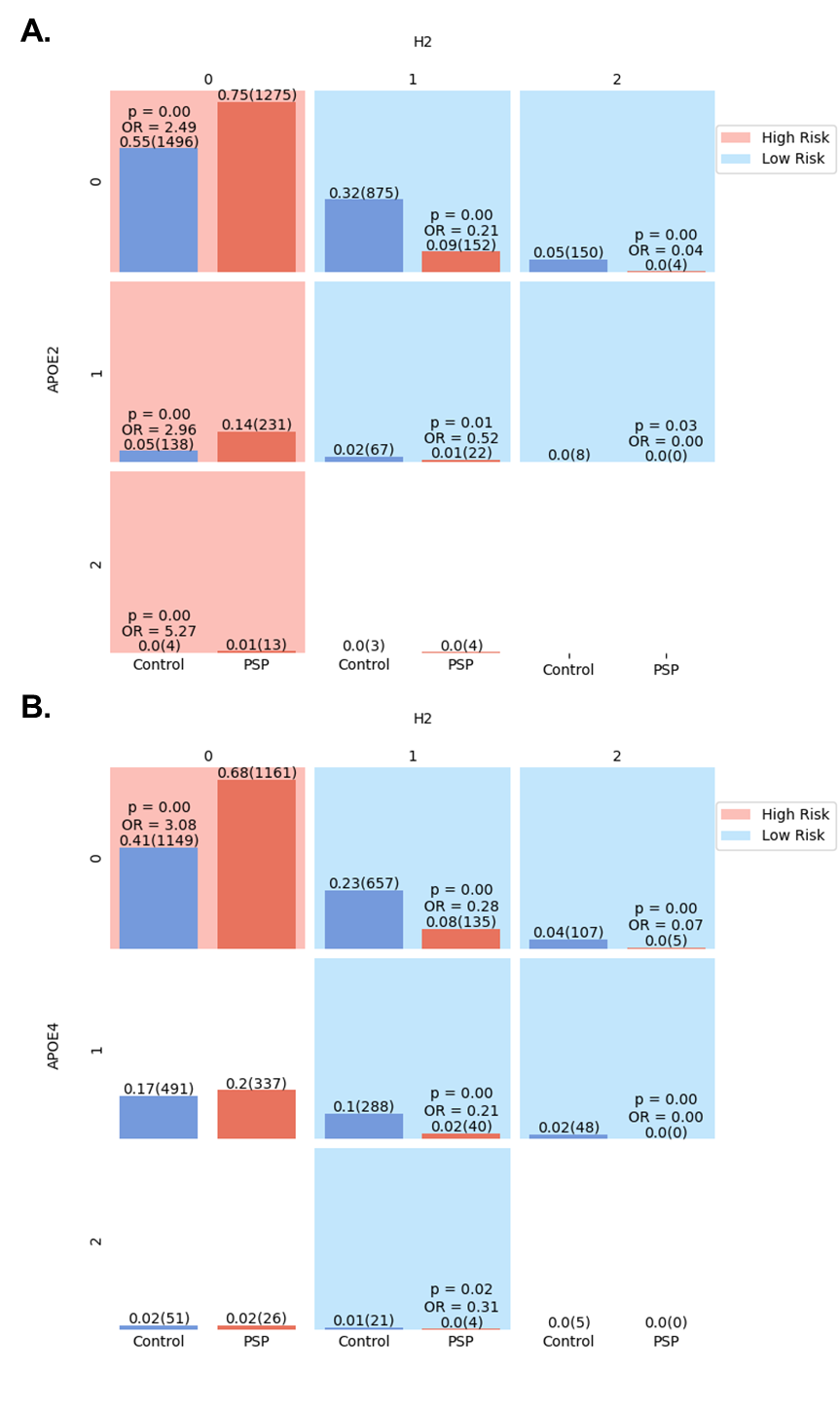


Figure S4. Visualization of the addictive risk between APOE4/2 and H1/H2 haplotype in PSP.

**A.** APOE2 and H1/H2 haplotype. **B.** APOE3 and H1/H2 haplotype. The ratio of case and control in each cell is shown. Cells with significantly higher/lower cases than controls by fisher’s exact test are marked red/blue.


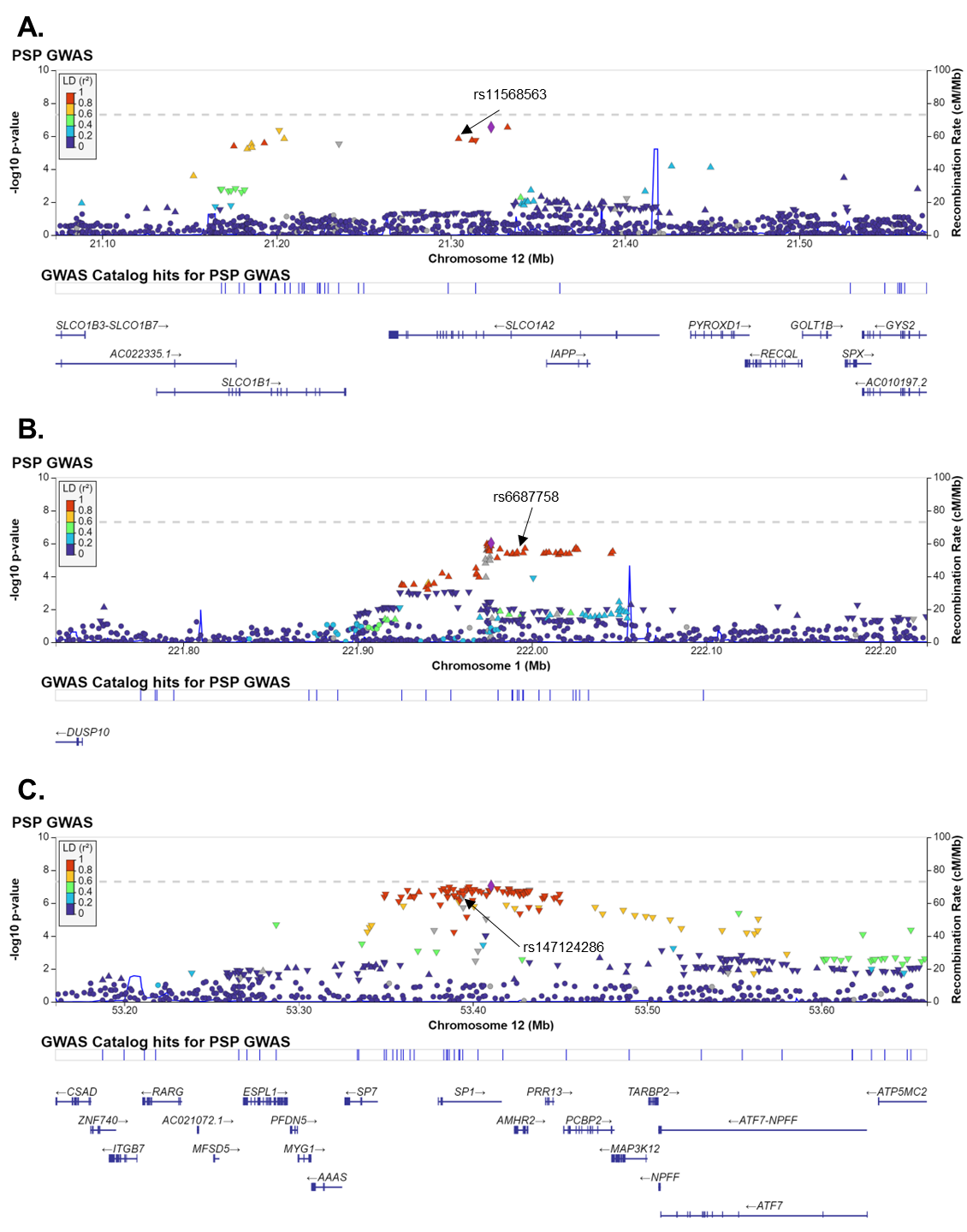


Figure S5. Suggestive signals for *SLCO1A2,* *DUSP10*, and *SP1*.

**A.** Significant signals in *SCLO1A2*. The arrow is pointed to the genome-wide significant signal in previous study. **B.** Significant signals in *DUSP10*. The arrow is pointed to the genome-wide significant signal in previous study. **C.** Significant signals in *SP1* region. The arrow is pointed to the suggestive signal (grey triangle) in previous study. LD is calculated on 1000G Europeans.


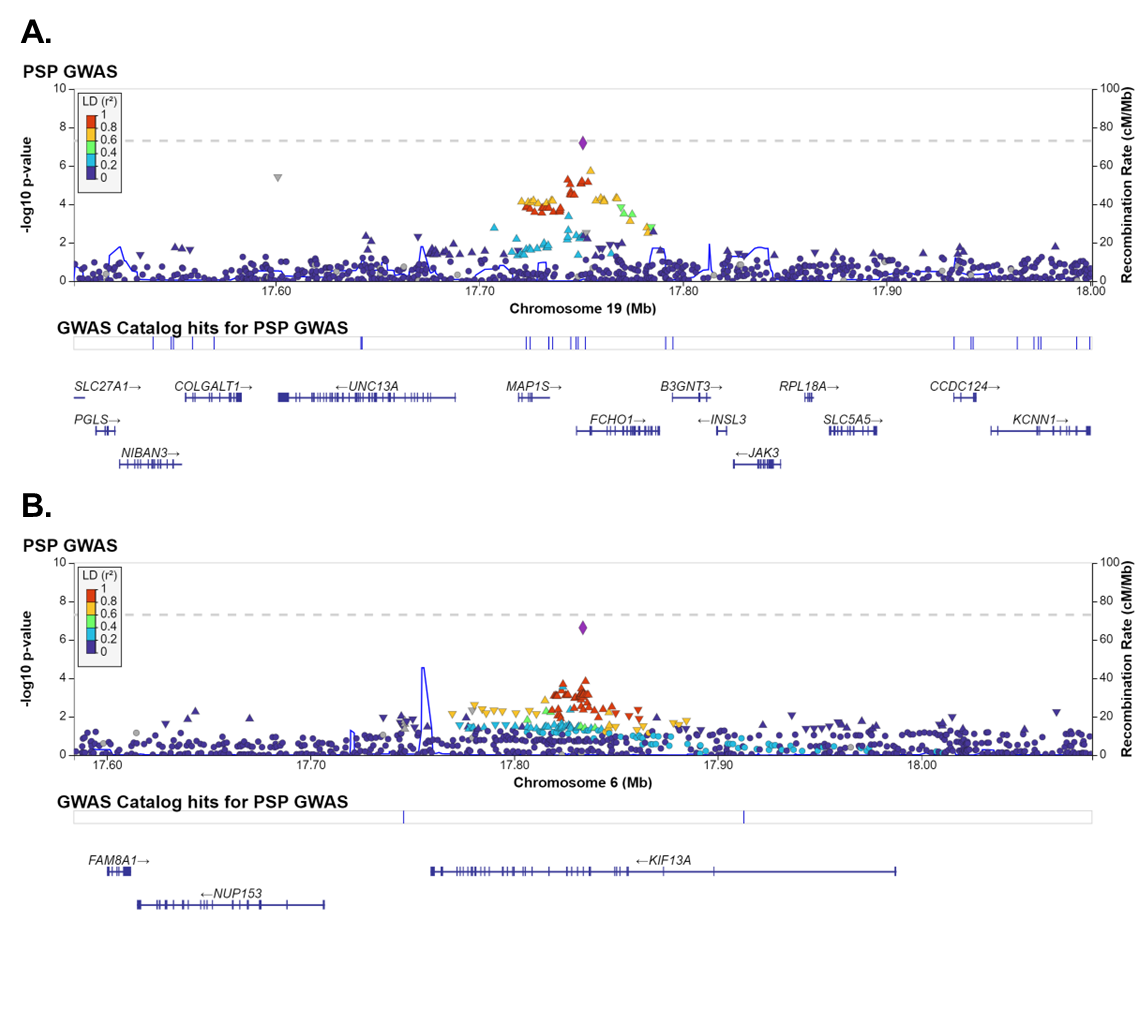


Figure S6. Suggestive signals for *FCHO1*/*MAP1S* and *KIF13A*.

**A.** Significant signals in *FCHO1*/*MAP1S*. **B.** Significant signals in *KIF13A*. LD is calculated on 1000G Europeans.


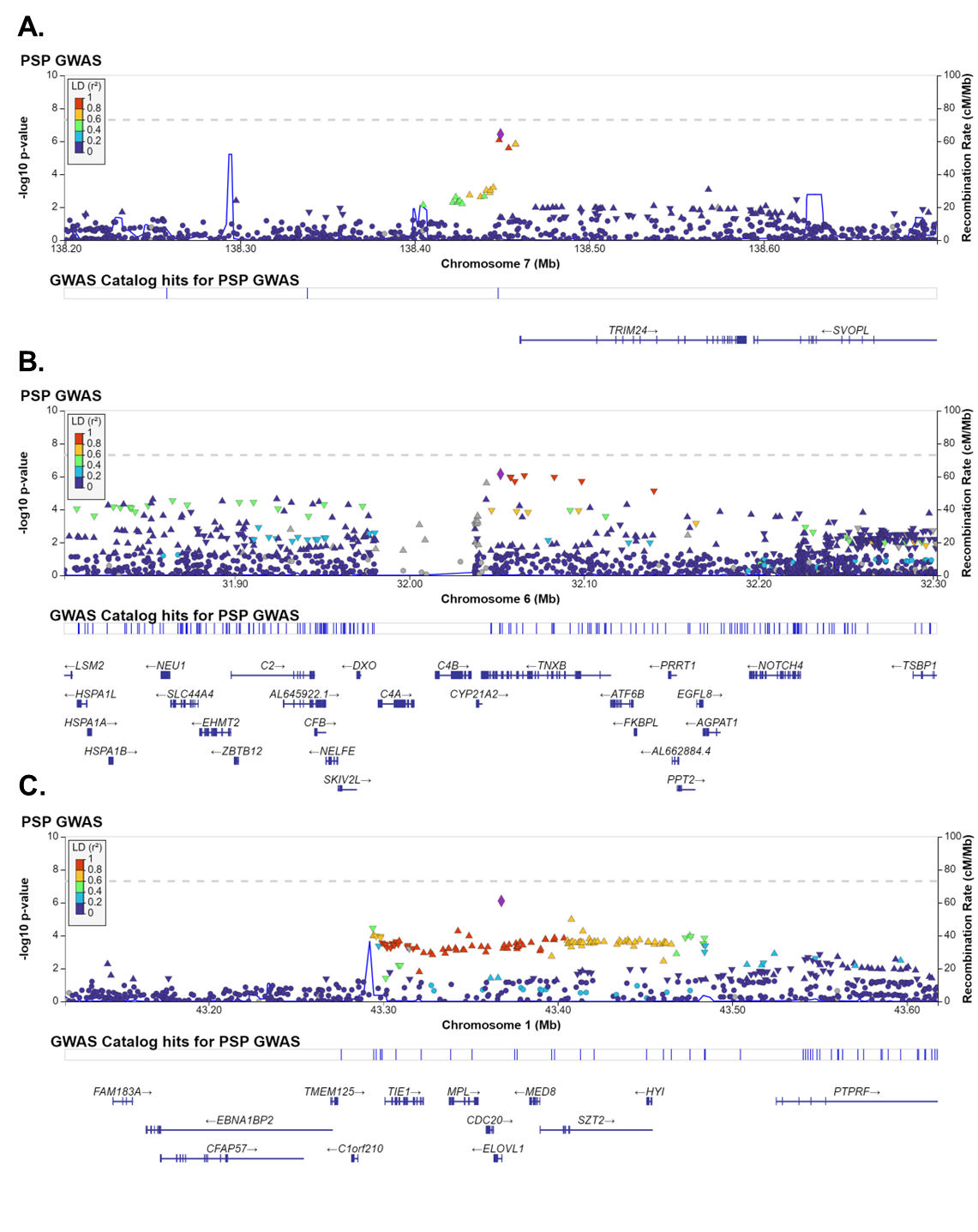


Figure S7. Suggestive signals for *TRIM24*, *TNXB*, and *ELOVL1*.

**A.** Significant signals in *TRIM24*. **B.** Significant signals in *TNXB*. **C.** Significant signals in *ELOVL1*. LD is calculated on 1000G Europeans.


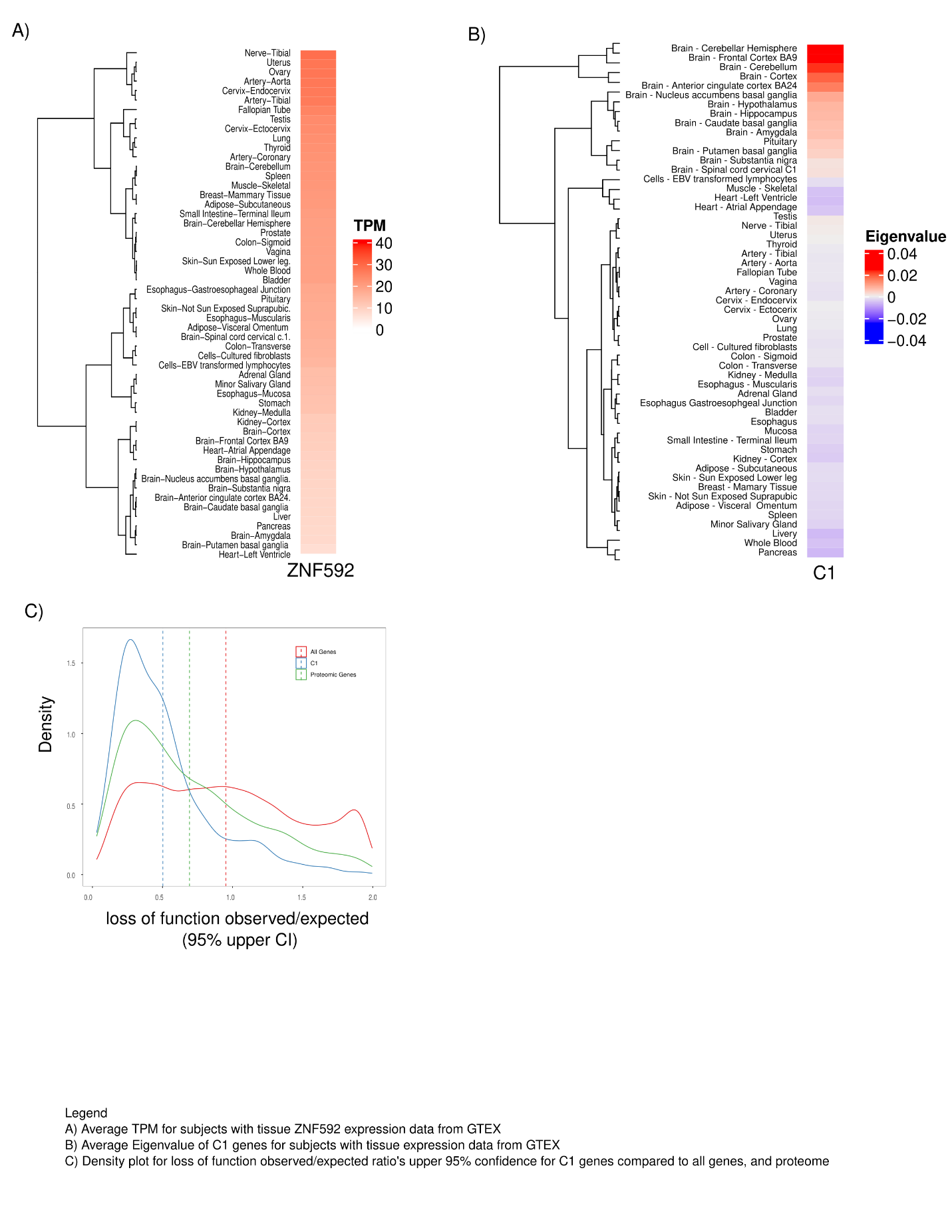


Figure S8. Functional analysis of *ZNF592* and the C1 module.

**A.** Average TPM for subjects with tissue ZNF592 expression data from GTEx. **B.** Average eigenvalue of C1 genes for subjects with tissue expression data from GTEx. **C.** Density plot for loss of function observed/expected ratio’s upper 95% confidence for C1 genes compared to all genes, and proteome.


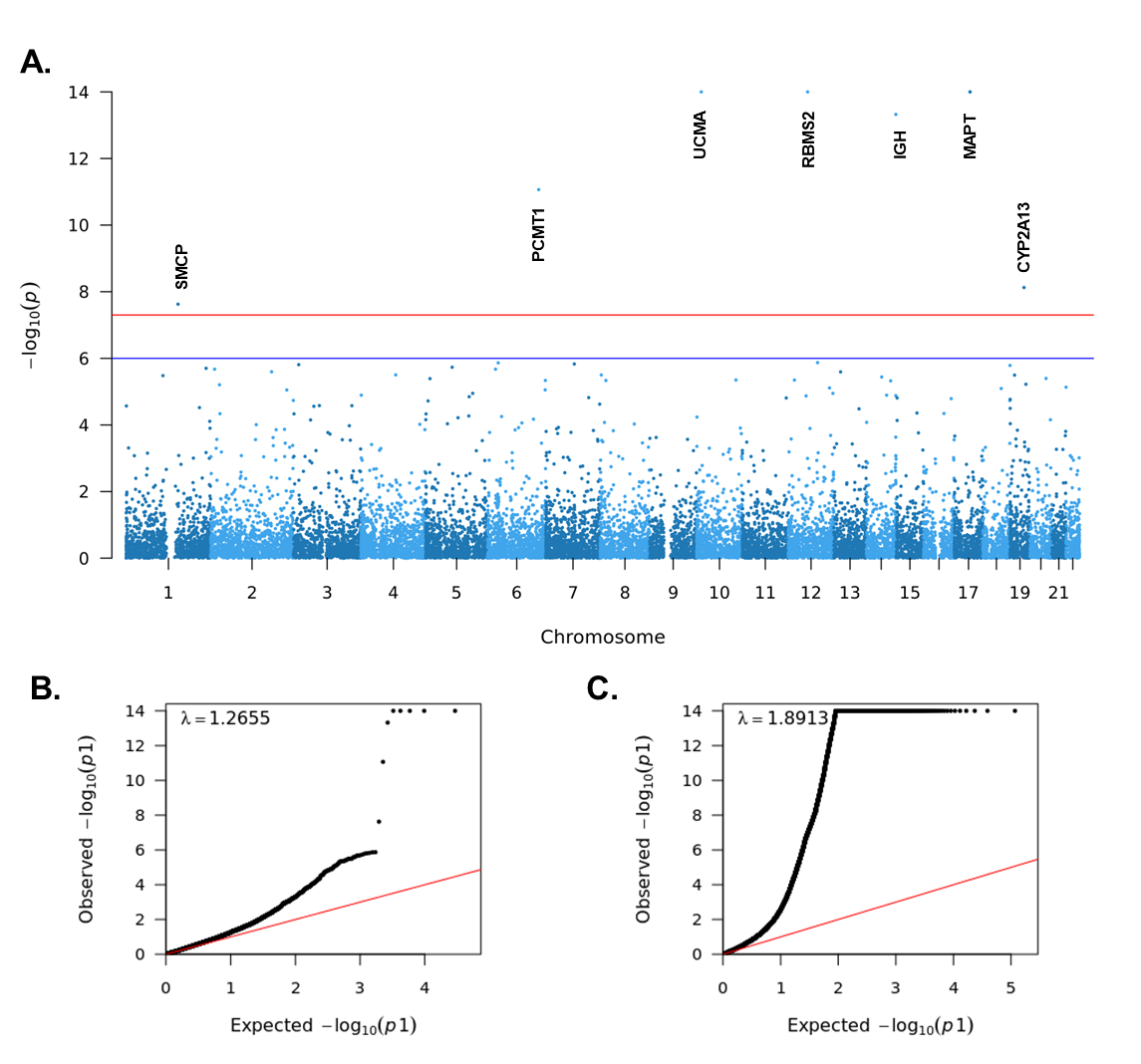


Figure S9. Genome-wide association analysis for structural variants.

**A.** Manhattan plot for high-quality structural variants. Variants with a P value below 1 × 10^-14^ are not shown. Loci with a suggestive or genome-wide significant signal are annotated. The red horizontal line represents genome-wide significance level (5 × 10-8). The blue horizontal line represents suggestive significance level (1 × 10-6). **B.** Q-Q plot for the high-quality structural variants. **C.** Q-Q plot for all structural variants.


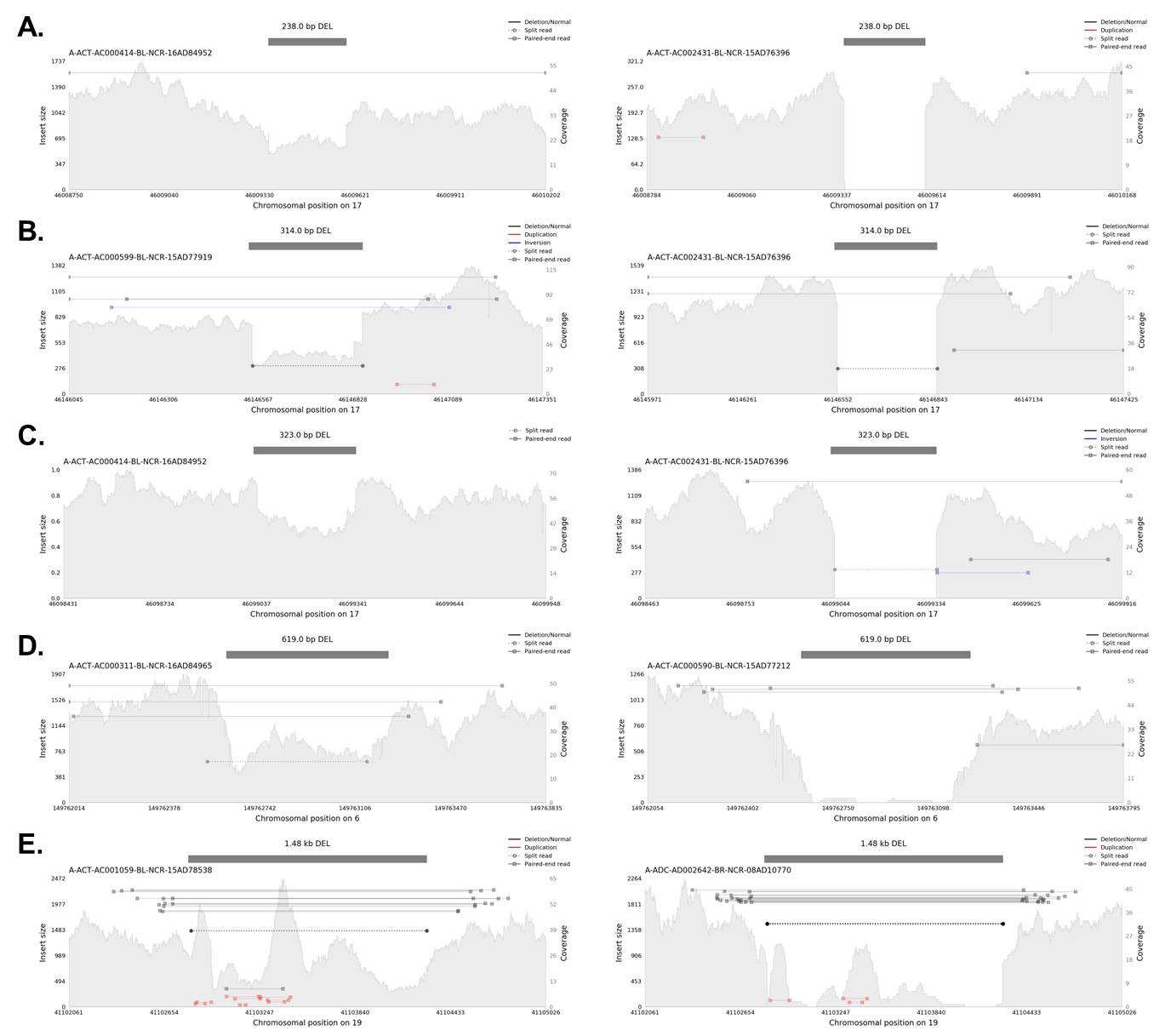


Figure S10. Samplot for genome-wide significant deletions.

**A-E.** Samplot of heterozygous/homozygous deletion for chr17:46009357-46009595:DEL, chr17:46146541-46146855:DEL, chr17:46099028-46099351:DEL, chr6:149762615-149763234:DEL, and chr19:41102802-41104285:DEL.


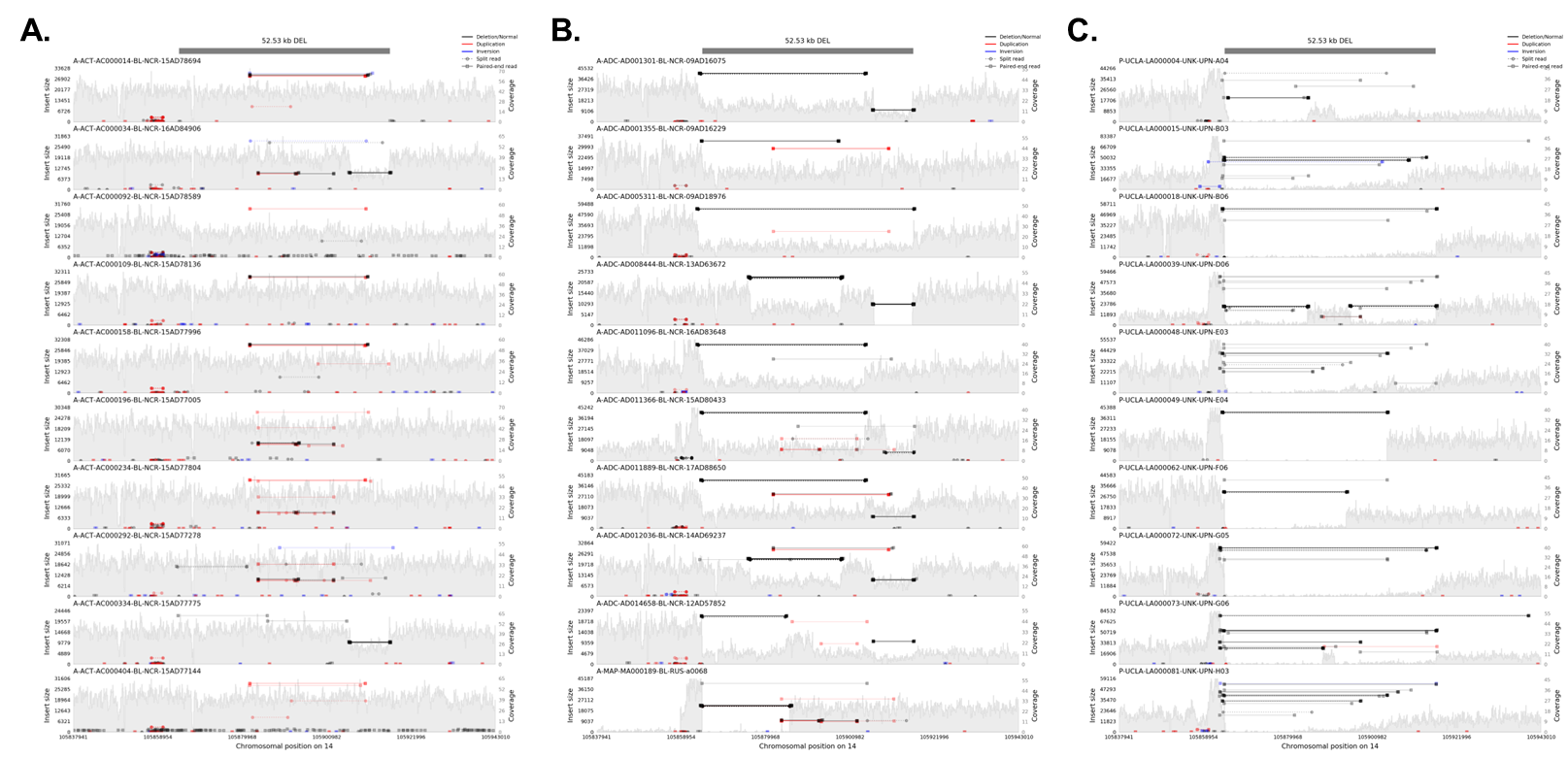


Figure S11. Deletions in immunoglobulin heavy locus (IGH).

**A.** Samples without deletion. **B.** Sample with heterozygous deletion **C.** Samples with homozygous deletion.


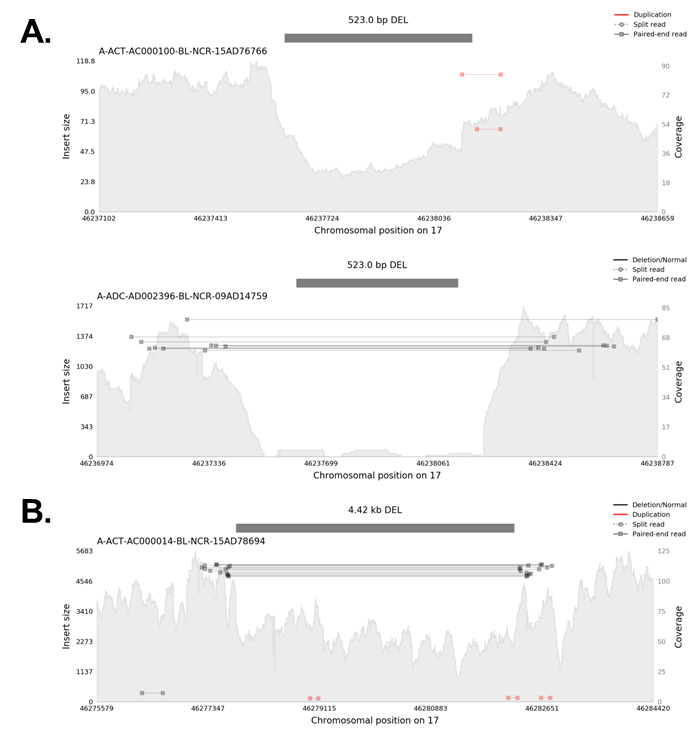


Figure S12. Samplot for common deletions and duplications in the H1/H2 region.

**A.** Samplot of heterozygous/homozygous deletion (chr17:46237619-46238142:DEL). **B.** Samplot of heterozygous deletion for chr17:46277789-46282210:DEL.


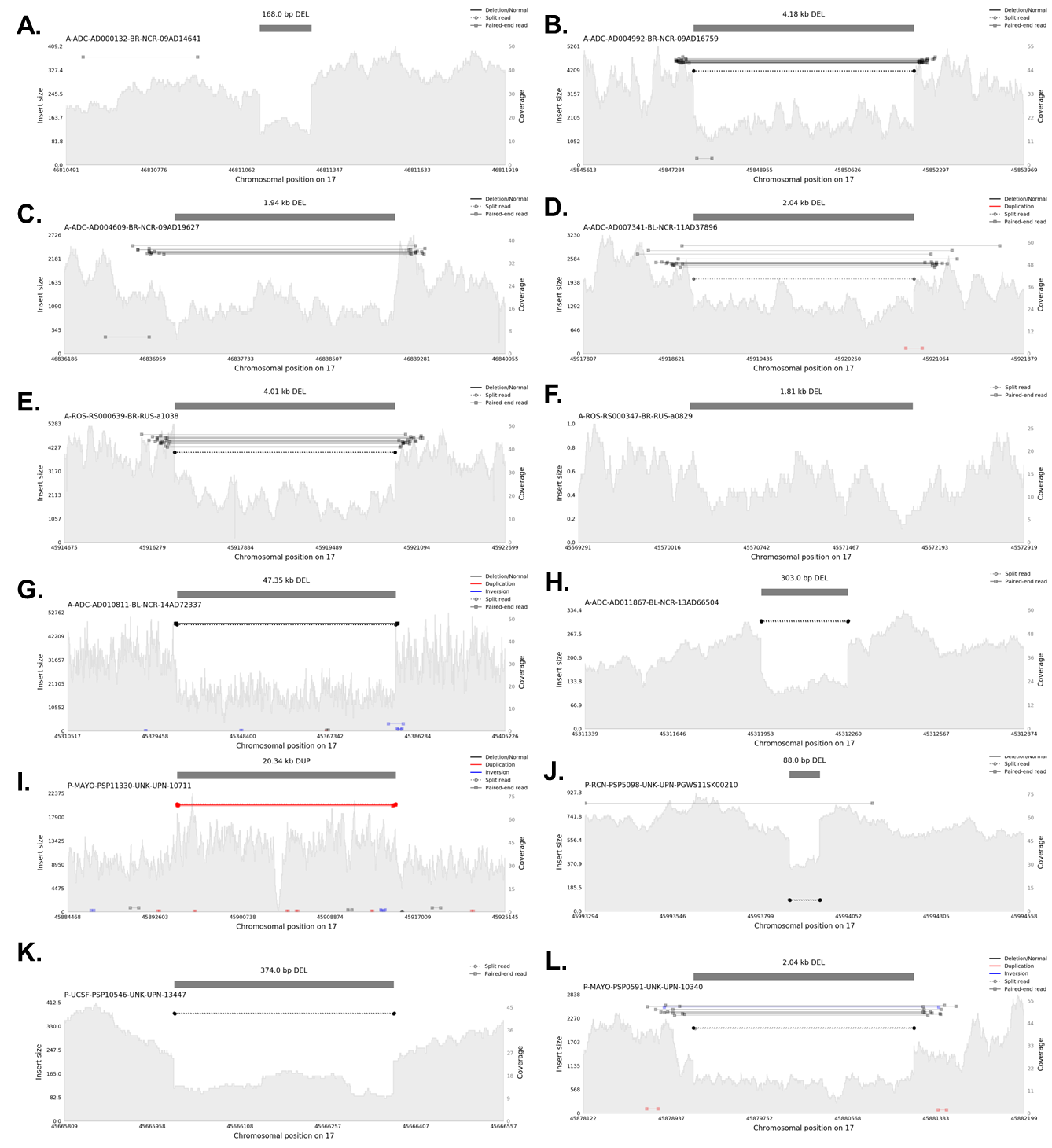


Figure S13. Samplot for rare deletions and duplications in the H1/H2 region.

**A-L.** Samplot for one sample have the deletion/duplication (chr17:46811121-46811289:DEL, chr17:45847702-45851880:DEL, chr17:46837153-46839088:DEL, chr17:45918825-45920861:DEL, chr17:45916681-45920693:DEL, chr17:45570198-45572012:DEL, chr17:45334194-45381549:DEL, chr17:45311955-45312258:DEL, chr17:45894637-45914976:DUP, chr17:45993882-45993970:DEL, chr17:45665996-45666370:DEL, chr17:45879141-45881180:DEL).


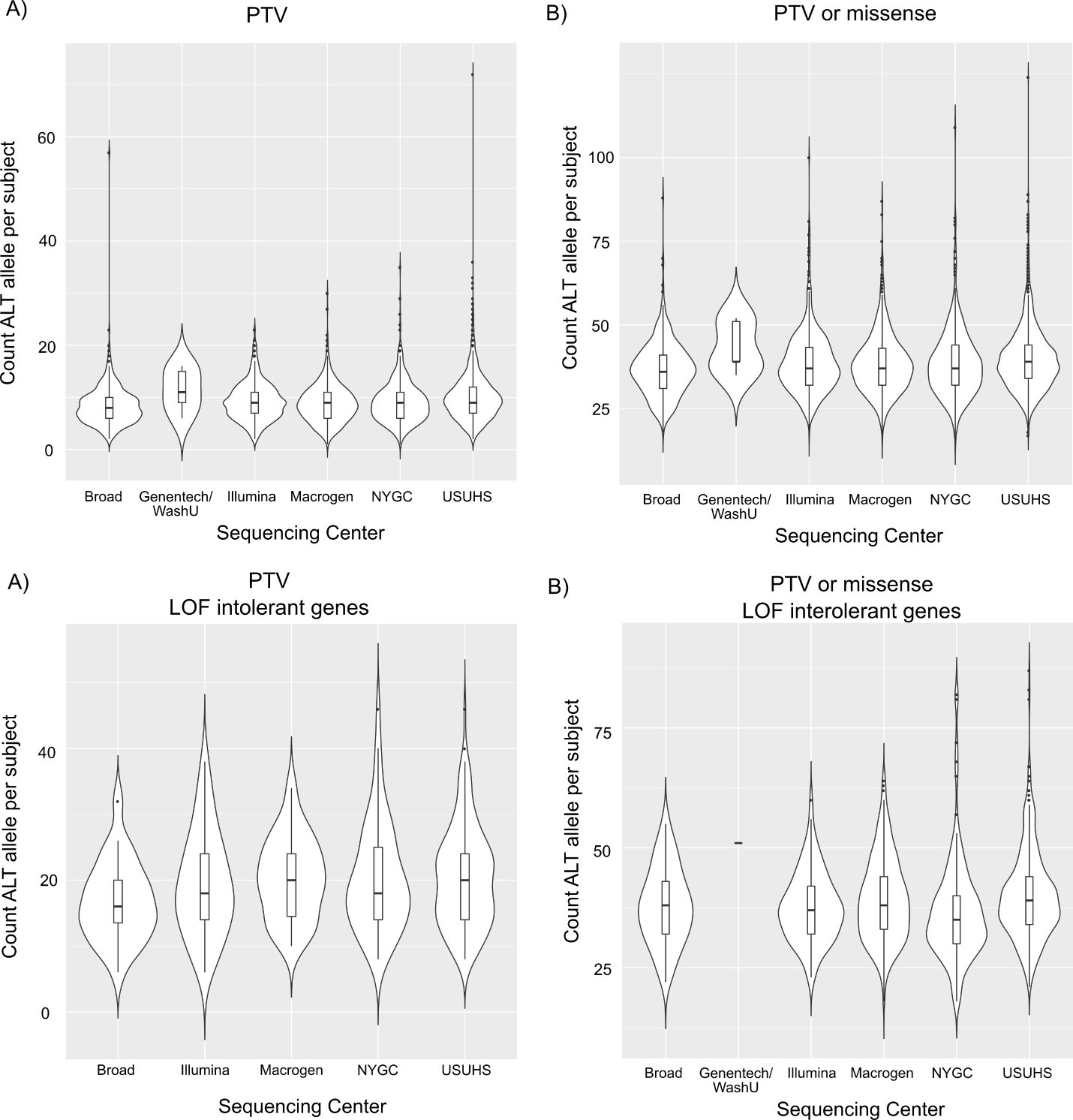


Figure S14. Counts of ALT alleles by sequencing center.

**A.** Counts of ALT alleles in protein truncating variants for subjects stratified by sequencing center. **B.** Counts of ALT alleles in protein truncating variants or damaging missense variants for subjects stratified by sequencing center. **C.** Counts of ALT alleles in protein truncating variants in loss of function intolerant genes (LOEUF ≤ 0.35) for subjects stratified by sequencing center. **D.** Counts of ALT alleles in protein truncating variants or damaging missense variants in loss of function intolerant genes (LOEUF ≤ 0.35) for subjects stratified by sequencing center.

Supplementary Methods

Cohorts with selection bias against APOE ε2/ε4

The ADSP-FUS1-APOEextremes study used an age extremes sampling approach stratified by *APOE* genotype, comparing younger onset AD cases against older cognitively normal controls: the controls were *APOE* ε4/ε4 controls with age-at-last-assessment ≥ 75 years, *APOE* ε3/ε4 controls with age-at-last-assessment ≥ 80 years, or *APOE* ε3/ε3 controls with age-at-last-assessment ≥ 85 years[1]. The ADSP-FUS1-StEPAD1 study aims to identify and characterize novel genetic variants that promote resilience to AD pathology in the presence of the *APOE* ε4 allele: controls from ADSP-FUS1-StEPAD1 were protected *APOE* ε4 carriers have normal cognition at older age[1]. The CacheCounty study selects “AD resilient individuals” and define them as individuals who are at least 75 years old, cognitively normal, and carry at least one *APOE* ε4 allele[2].

APOE genotype validation

For the genotyping reaction, 9.2 ng of genomic DNA was combined with TaqMan Master Mix (Applied Biosystems, Thermo Fisher Part No. 4371357) and 40X TaqMan assay in a total volume of 5ul. Two assays were performed: rs429358 (C___3084793_20, VIC=C, FAM=T, Applied Biosystems, Thermo Fisher) and rs7412 (C____904973_10, VIC=C, FAM=T, Applied Biosystems, Thermo Fisher). The DNA was amplified in a GeneAmp PCR System 9700 instrument (Applied Biosystems, Thermo Fisher) in a 384 well format with the following program: 50°C 2min, 95°C 10min followed by 40 cycles of 95°C 15sec, 60°C 1min. The amplified products were visualized using the QuantStudio 12K Flex instrument (Applied Biosystems) and the genotypes were analyzed using the TaqMan Genotyper software (Version 1.3) (Applied Biosystems, Thermo Fisher). For samples that were discordant between the WGS and TaqMan genotype or failed TaqMan genotyping, the APOE genotype was confirmed with Sanger Sequencing. Genomic DNA (~50ng) was amplified using a SimpliAmp Thermal Cycler (Applied Biosystems, Thermo Fisher) in a 20ul reaction volume with HotStarTaq Master Mix (Qiagen) in the presence of 2uM primers (Forward=5’-CTGTCTCTGTCTCCTTCTCTCGG-3’ and Reverse=5’ ACCTGCTCCTTCACCTCGTC-3’, IDT). The PCR conditions used were: 95°C 15min followed by 30 cycles of 95°C 20sec, 55°C 30sec, 72°C 2min with a final extension of 72°C 7min. The amplified PCR products were prepared for Sanger sequencing by adding ExoSAP-IT (USB) and incubating at 37°C for 45min followed by 80°C for 15min. The PCR products were then Sanger sequenced using the BigDye® Terminator v3.1 Cycle Sequencing kit (Part No. 4336917 Applied Biosystems, Thermo Fisher). The sequencing reaction contained BigDye® Terminator v3.1 Ready Reaction Mix, 5X Sequencing Buffer, 5M Betaine solution (Part No. B0300 Sigma) and 0.64uM sequencing primer (IDT) in a total volume of 5ul. The sequencing reaction was performed in a SimpliAmp Thermal Cycler (Applied Biosystems, Thermo Fisher) using the following program: 96°C 1min followed by 25 cycles of 96°C 10sec, 50°C 5sec, 60°C 1min15sec. The products were cleaned using XTerminator and SAM Solution as directed (Part No. 4376487, Applied Biosystems, Thermo Fisher) with 30min of shaking at 1800rpm followed by centrifugation at 1000 rpm for 2min. The sequencing products were analyzed on a 3130xl Genetic Analyzer (Applied Biosystems, Thermo Fisher) and the sequencing traces were analyzed using Sequencher 5.4 (Gene Code).

Masked regions in genome

There are regions in the human genome that tend to have anomalous, or high signal in WGS experiments[3]. SVs that reside in those regions can be unreliable and should be reported. Specifically, we compiled problematic regions in the genome from the following sources: (1) the ENCODE blacklist: a comprehensive set of regions that could result in erroneous signal[4]; (2) the 1000 Genome masks: regions of the genome that are more or less accessible to next generation sequencing methods using short reads; (3) the set of assembly gaps defined by UCSC; (4) the set of segmental duplications defined by UCUC; (5) the low-complexity regions, satellite sequences and simple repeats defined by RepeatMasker[5].

Aggregated test for rare variants

Multi-allelic variants were split into biallelic variants. Variants where ALT=*, representing a spanning deletion, were removed. Bi-allelic and multi-allelic variants were concatenated, and duplicated variants were removed. Variants were removed if they were monomorphic, did not pass VQSR, had an average read depth ≥ 500, or if all calls have DP<10 & GQ<20. Individual calls with a DP<10 or GQ<20 were set to missing. Indels were left aligned using the GRCh38 reference[6,7]. Then, variants with a missing rate > 0.1 or a *P*_HWE_ < 1 × 10^-7^ in controls were removed, resulting in 91,863,622 variants.

After LD clumping with a r^2^ cutoff of 0.2, we applied the bigsnpr R package to perform PCA using variants with MAF > 1%. We tested if genes with PTVs or PTVs/missense variants were associated with PSP using the sequence kernel association test-optimized (SKAT-O)[8] (SKAT R package version 2.0.1)[9]. We used a linear kernel and weighed each variant by the maximum external database MAF where lower MAF would have higher weight. We normalized variant MAFs, where ${MAF}_{norm}={MAF}_{ext}/{MAF}_{max}$, where ${MAF}_{ext}$ is the external database MAF from gnomAD[10], and ${MAF}_{max}=0.0001$. The variant weight is defined by the ${MAF}_{norm}$ on the β (1,4) distribution. Thus, variant weight is high at very low ${MAF}_{norm}$ and spread across the range of 1 to 4. Covariates included sex, PC1-3, and H1/H2 haplotype.

References

1. Beecham GW, Bis JC, Martin ER, Choi SH, DeStefano AL, Van Duijn CM, et al. The Alzheimer’s Disease Sequencing Project: study design and sample selection. Neurology Genetics. 2017;3(5).

2. for the Alzheimer’s Disease Neuroimaging Initiative, Ridge PG, Karch CM, Hsu S, Arano I, Teerlink CC, et al. Linkage, whole genome sequence, and biological data implicate variants in RAB10 in Alzheimer’s disease resilience. Genome Med. 2017 Dec;9(1):100.

3. Scherer SW, Lee C, Birney E, Altshuler DM, Eichler EE, Carter NP, et al. Challenges and standards in integrating surveys of structural variation. Nat Genet. 2007 Jul;39(7):S7–15.

4. Amemiya HM, Kundaje A, Boyle AP. The ENCODE blacklist: identification of problematic regions of the genome. Scientific reports. 2019;9(1):1–5.

5. Smit, AFA, Hubley, R & Green, P. RepeatMasker Open-4.0. 2013-2015 <http://www.repeatmasker.org>.

6. Genome Reference Consortium. GRCh38 reference 000001405.15 [Internet]. [cited 2022 Jun 22]. Available from: https://ftp.ncbi.nlm.nih.gov/genomes/all/GCA/000/001/405/GCA_000001405.15_GRCh38/seqs_for_alignment_pipelines.ucsc_ids/GCA_000001405.15_GRCh38_no_alt_analysis_set.fna.gz

7. Schneider VA, Graves-Lindsay T, Howe K, Bouk N, Chen HC, Kitts PA, et al. Evaluation of GRCh38 and de novo haploid genome assemblies demonstrates the enduring quality of the reference assembly. Genome Res. 2017 May;27(5):849–64.

8. Lee S, Emond MJ, Bamshad MJ, Barnes KC, Rieder MJ, Nickerson DA, et al. Optimal Unified Approach for Rare-Variant Association Testing with Application to Small-Sample Case-Control Whole-Exome Sequencing Studies. Am J Hum Genet. 2012 Aug 10;91(2):224–37.

9. Seunggeun Lee, Zhangchen Zhao, Larisa Miropolsky, Michael Wu. SKAT: SNP-Set (Sequence) Kernel Association Test [Internet]. 2020. Available from: https://CRAN.R-project.org/package=SKAT

10. Karczewski KJ, Francioli LC, Tiao G, Cummings BB, Alföldi J, Wang Q, et al. The mutational constraint spectrum quantified from variation in 141,456 humans. Nature. 2020 May;581(7809):434–43.

Acknowledgements

### ADSP (sa000001) data:

The Alzheimer’s Disease Sequencing Project (ADSP) is comprised of two Alzheimer’s Disease (AD) genetics consortia and three National Human Genome Research Institute (NHGRI) funded Large Scale Sequencing and Analysis Centers (LSAC). The two AD genetics consortia are the Alzheimer’s Disease Genetics Consortium (ADGC) funded by NIA (U01 AG032984), and the Cohorts for Heart and Aging Research in Genomic Epidemiology (CHARGE) funded by NIA (R01 AG033193), the National Heart, Lung, and Blood Institute (NHLBI), other National Institute of Health (NIH) institutes and other foreign governmental and non-governmental organizations. The Discovery Phase analysis of sequence data is supported through UF1AG047133 (to Drs. Schellenberg, Farrer, Pericak-Vance, Mayeux, and Haines); U01AG049505 to Dr. Seshadri; U01AG049506 to Dr. Boerwinkle; U01AG049507 to Dr. Wijsman; and U01AG049508 to Dr. Goate and the Discovery Extension Phase analysis is supported through U01AG052411 to Dr. Goate, U01AG052410 to Dr. Pericak-Vance and U01 AG052409 to Drs. Seshadri and Fornage.

Sequencing for the Follow Up Study (FUS) is supported through U01AG057659 (to Drs. PericakVance, Mayeux, and Vardarajan) and U01AG062943 (to Drs. Pericak-Vance and Mayeux). Data generation and harmonization in the Follow-up Phase is supported by U54AG052427 (to Drs. Schellenberg and Wang). The FUS Phase analysis of sequence data is supported through U01AG058589 (to Drs. Destefano, Boerwinkle, De Jager, Fornage, Seshadri, and Wijsman), U01AG058654 (to Drs. Haines, Bush, Farrer, Martin, and Pericak-Vance), U01AG058635 (to Dr. Goate), RF1AG058066 (to Drs. Haines, Pericak-Vance, and Scott), RF1AG057519 (to Drs. Farrer and Jun), R01AG048927 (to Dr. Farrer), and RF1AG054074 (to Drs. Pericak-Vance and Beecham).

The ADGC cohorts include: Adult Changes in Thought (ACT) (U01 AG006781, U19 AG066567), the Alzheimer’s Disease Research Centers (ADRC) (P30 AG062429, P30 AG066468, P30 AG062421, P30 AG066509, P30 AG066514, P30 AG066530, P30 AG066507, P30 AG066444, P30 AG066518, P30 AG066512, P30 AG066462, P30 AG072979, P30 AG072972, P30 AG072976, P30 AG072975, P30 AG072978, P30 AG072977, P30 AG066519, P30 AG062677, P30 AG079280, P30 AG062422, P30 AG066511, P30 AG072946, P30 AG062715, P30 AG072973, P30 AG066506, P30 AG066508, P30 AG066515, P30 AG072947, P30 AG072931, P30 AG066546, P20 AG068024, P20 AG068053, P20 AG068077, P20 AG068082, P30 AG072958, P30 AG072959), the Chicago Health and Aging Project (CHAP) (R01 AG11101, RC4 AG039085, K23 AG030944), Indiana Memory and Aging Study (IMAS) (R01 AG019771), Indianapolis Ibadan (R01 AG009956, P30 AG010133), the Memory and Aging Project (MAP) ( R01 AG17917), Mayo Clinic (MAYO) (R01 AG032990, U01 AG046139, R01 NS080820, RF1 AG051504, P50 AG016574), Mayo Parkinson’s Disease controls (NS039764, NS071674, 5RC2HG005605), University of Miami (R01 AG027944, R01 AG028786, R01 AG019085, IIRG09133827, A2011048), the Multi-Institutional Research in Alzheimer’s Genetic Epidemiology Study (MIRAGE) (R01 AG09029, R01 AG025259), the National Centralized Repository for Alzheimer’s Disease and Related Dementias (NCRAD) (U24 AG021886), the National Institute on Aging Late Onset Alzheimer’s Disease Family Study (NIA- LOAD) (U24 AG056270), the Religious Orders Study (ROS) (P30 AG10161, R01 AG15819), the Texas Alzheimer’s Research and Care Consortium (TARCC) (funded by the Darrell K Royal Texas Alzheimer’s Initiative), Vanderbilt University/Case Western Reserve University (VAN/CWRU) (R01 AG019757, R01 AG021547, R01 AG027944, R01 AG028786, P01 NS026630, and Alzheimer’s Association), the Washington Heights-Inwood Columbia Aging Project (WHICAP) (RF1 AG054023), the University of Washington Families (VA Research Merit Grant, NIA: P50AG005136, R01AG041797, NINDS: R01NS069719), the Columbia University Hispanic Estudio Familiar de Influencia Genetica de Alzheimer (EFIGA) (RF1 AG015473), the University of Toronto (UT) (funded by Wellcome Trust, Medical Research Council, Canadian Institutes of Health Research), and Genetic Differences (GD) (R01 AG007584). The CHARGE cohorts are supported in part by National Heart, Lung, and Blood Institute (NHLBI) infrastructure grant HL105756 (Psaty), RC2HL102419 (Boerwinkle) and the neurology working group is supported by the National Institute on Aging (NIA) R01 grant AG033193.

The CHARGE cohorts participating in the ADSP include the following: Austrian Stroke Prevention Study (ASPS), ASPS-Family study, and the Prospective Dementia Registry-Austria (ASPS/PRODEM-Aus), the Atherosclerosis Risk in Communities (ARIC) Study, the Cardiovascular Health Study (CHS), the Erasmus Rucphen Family Study (ERF), the Framingham Heart Study (FHS), and the Rotterdam Study (RS). ASPS is funded by the Austrian Science Fond (FWF) grant number P20545-P05 and P13180 and the Medical University of Graz. The ASPS-Fam is funded by the Austrian Science Fund (FWF) project I904), the EU Joint Programme – Neurodegenerative Disease Research (JPND) in frame of the BRIDGET project (Austria, Ministry of Science) and the Medical University of Graz and the Steiermärkische Krankenanstalten Gesellschaft. PRODEM-Austria is supported by the Austrian Research Promotion agency (FFG) (Project No. 827462) and by the Austrian National Bank (Anniversary Fund, project 15435. ARIC research is carried out as a collaborative study supported by NHLBI contracts (HHSN268201100005C, HHSN268201100006C, HHSN268201100007C, HHSN268201100008C, HHSN268201100009C, HHSN268201100010C, HHSN268201100011C, and HHSN268201100012C). Neurocognitive data in ARIC is collected by U01 2U01HL096812, 2U01HL096814, 2U01HL096899, 2U01HL096902, 2U01HL096917 from the NIH (NHLBI, NINDS, NIA and NIDCD), and with previous brain MRI examinations funded by R01-HL70825 from the NHLBI. CHS research was supported by contracts HHSN268201200036C, HHSN268200800007C, N01HC55222, N01HC85079, N01HC85080, N01HC85081, N01HC85082, N01HC85083, N01HC85086, and grants U01HL080295 and U01HL130114 from the NHLBI with additional contribution from the National Institute of Neurological Disorders and Stroke (NINDS). Additional support was provided by R01AG023629, R01AG15928, and R01AG20098 from the NIA. FHS research is supported by NHLBI contracts N01-HC-25195 and HHSN268201500001I. This study was also supported by additional grants from the NIA (R01s AG054076, AG049607 and AG033040 and NINDS (R01 NS017950). The ERF study as a part of EUROSPAN (European Special Populations Research Network) was supported by European Commission FP6 STRP grant number 018947 (LSHG-CT-2006-01947) and also received funding from the European Community’s Seventh Framework Programme (FP7/2007-2013)/grant agreement HEALTH-F4- 2007-201413 by the European Commission under the programme “Quality of Life and Management of the Living Resources” of 5th Framework Programme (no. QLG2-CT-2002- 01254). High-throughput analysis of the ERF data was supported by a joint grant from the Netherlands Organization for Scientific Research and the Russian Foundation for Basic Research (NWO-RFBR 047.017.043). The Rotterdam Study is funded by Erasmus Medical Center and Erasmus University, Rotterdam, the Netherlands Organization for Health Research and Development (ZonMw), the Research Institute for Diseases in the Elderly (RIDE), the Ministry of Education, Culture and Science, the Ministry for Health, Welfare and Sports, the European Commission (DG XII), and the municipality of Rotterdam. Genetic data sets are also supported by the Netherlands Organization of Scientific Research NWO Investments (175.010.2005.011, 911-03-012), the Genetic Laboratory of the Department of Internal Medicine, Erasmus MC, the Research Institute for Diseases in the Elderly (014-93-015; RIDE2), and the Netherlands Genomics Initiative (NGI)/Netherlands Organization for Scientific Research (NWO) Netherlands Consortium for Healthy Aging (NCHA), project 050-060-810. All studies are grateful to their participants, faculty and staff. The content of these manuscripts is solely the responsibility of the authors and does not necessarily represent the official views of the National Institutes of Health or the U.S. Department of Health and Human Services.

The FUS cohorts include: the Alzheimer’s Disease Research Centers (ADRC) (P30 AG062429, P30 AG066468, P30 AG062421, P30 AG066509, P30 AG066514, P30 AG066530, P30 AG066507, P30 AG066444, P30 AG066518, P30 AG066512, P30 AG066462, P30 AG072979, P30 AG072972, P30 AG072976, P30 AG072975, P30 AG072978, P30 AG072977, P30 AG066519, P30 AG062677, P30 AG079280, P30 AG062422, P30 AG066511, P30 AG072946, P30 AG062715, P30 AG072973, P30 AG066506, P30 AG066508, P30 AG066515, P30 AG072947, P30 AG072931, P30 AG066546, P20 AG068024, P20 AG068053, P20 AG068077, P20 AG068082, P30 AG072958, P30 AG072959), Alzheimer’s Disease Neuroimaging Initiative (ADNI) (U19AG024904), Amish Protective Variant Study (RF1AG058066), Cache County Study (R01AG11380, R01AG031272, R01AG21136, RF1AG054052), Case Western Reserve University Brain Bank (CWRUBB) (P50AG008012), Case Western Reserve University Rapid Decline (CWRURD) (RF1AG058267, NU38CK000480), CubanAmerican Alzheimer’s Disease Initiative (CuAADI) (3U01AG052410), Estudio Familiar de Influencia Genetica en Alzheimer (EFIGA) (5R37AG015473, RF1AG015473, R56AG051876), Genetic and Environmental Risk Factors for Alzheimer Disease Among African Americans Study (GenerAAtions) (2R01AG09029, R01AG025259, 2R01AG048927), Gwangju Alzheimer and Related Dementias Study (GARD) (U01AG062602), Hillblom Aging Network (2014-A-004-NET, R01AG032289, R01AG048234), Hussman Institute for Human Genomics Brain Bank (HIHGBB) (R01AG027944, Alzheimer’s Association “Identification of Rare Variants in Alzheimer Disease”), Ibadan Study of Aging (IBADAN) (5R01AG009956), Longevity Genes Project (LGP) and LonGenity (R01AG042188, R01AG044829, R01AG046949, R01AG057909, R01AG061155, P30AG038072), Mexican Health and Aging Study (MHAS) (R01AG018016), Multi-Institutional Research in Alzheimer’s Genetic Epidemiology (MIRAGE) (2R01AG09029, R01AG025259, 2R01AG048927), Northern Manhattan Study (NOMAS) (R01NS29993), Peru Alzheimer’s Disease Initiative (PeADI) (RF1AG054074), Puerto Rican 1066 (PR1066) (Wellcome Trust (GR066133/GR080002), European Research Council (340755)), Puerto Rican Alzheimer Disease Initiative (PRADI) (RF1AG054074), Reasons for Geographic and Racial Differences in Stroke (REGARDS) (U01NS041588), Research in African American Alzheimer Disease Initiative (REAAADI) (U01AG052410), the Religious Orders Study (ROS) (P30 AG10161, P30 AG72975, R01 AG15819, R01 AG42210), the RUSH Memory and Aging Project (MAP) (R01 AG017917, R01 AG42210Stanford Extreme Phenotypes in AD (R01AG060747), University of Miami Brain Endowment Bank (MBB), University of Miami/Case Western/North Carolina A&T African American (UM/CASE/NCAT) (U01AG052410, R01AG028786), and Wisconsin Registry for Alzheimer’s Prevention (WRAP) (R01AG027161 and R01AG054047).

The four LSACs are: the Human Genome Sequencing Center at the Baylor College of Medicine (U54 HG003273), the Broad Institute Genome Center (U54HG003067), The American Genome Center at the Uniformed Services University of the Health Sciences (U01AG057659), and the Washington University Genome Institute (U54HG003079). Genotyping and sequencing for the ADSP FUS is also conducted at John P. Hussman Institute for Human Genomics (HIHG) Center for Genome Technology (CGT).

Biological samples and associated phenotypic data used in primary data analyses were stored at Study Investigators institutions, and at the National Centralized Repository for Alzheimer’s Disease and Related Dementias (NCRAD, U24AG021886) at Indiana University funded by NIA. Associated Phenotypic Data used in primary and secondary data analyses were provided by Study Investigators, the NIA funded Alzheimer’s Disease Centers (ADCs), and the National Alzheimer’s Coordinating Center (NACC, U24AG072122) and the National Institute on Aging Genetics of Alzheimer’s Disease Data Storage Site (NIAGADS, U24AG041689) at the University of Pennsylvania, funded by NIA. Harmonized phenotypes were provided by the ADSP Phenotype Harmonization Consortium (ADSP-PHC), funded by NIA (U24 AG074855, U01 AG068057 and R01 AG059716) and Ultrascale Machine Learning to Empower Discovery in Alzheimer’s Disease Biobanks (AI4AD, U01 AG068057). This research was supported in part by the Intramural Research Program of the National Institutes of health, National Library of Medicine. Contributors to the Genetic Analysis Data included Study Investigators on projects that were individually funded by NIA, and other NIH institutes, and by private U.S. organizations, or foreign governmental or nongovernmental organizations.

### ADNI (sa000002) data:

Data collection and sharing for this project was funded by the Alzheimer's Disease Neuroimaging Initiative (ADNI) (National Institutes of Health Grant U01 AG024904) and DOD ADNI (Department of Defense award number W81XWH-12-2-0012). ADNI is funded by the National Institute on Aging, the National Institute of Biomedical Imaging and Bioengineering, and through generous contributions from the following: AbbVie, Alzheimer’s Association; Alzheimer’s Drug Discovery Foundation; Araclon Biotech; BioClinica, Inc.; Biogen; Bristol-Myers Squibb Company; CereSpir, Inc.; Cogstate; Eisai Inc.; Elan Pharmaceuticals, Inc.; Eli Lilly and Company; EuroImmun; F. Hoffmann-La Roche Ltd and its affiliated company Genentech, Inc.; Fujirebio; GE Healthcare; IXICO Ltd.; Janssen Alzheimer Immunotherapy Research & Development, LLC.; Johnson & Johnson Pharmaceutical Research & Development LLC.; Lumosity; Lundbeck; Merck & Co., Inc.; Meso Scale Diagnostics, LLC.; NeuroRx Research; Neurotrack Technologies; Novartis Pharmaceuticals Corporation; Pfizer Inc.; Piramal Imaging; Servier; Takeda Pharmaceutical Company; and Transition Therapeutics. The Canadian Institutes of Health Research is providing funds to support ADNI clinical sites in Canada. Private sector contributions are facilitated by the Foundation for the National Institutes of Health (www.fnih.org). The grantee organization is the Northern California Institute for Research and Education, and the study is coordinated by the Alzheimer’s Therapeutic Research Institute at the University of Southern California. ADNI data are disseminated by the Laboratory for Neuro Imaging at the University of Southern California.

Additional information to include in an acknowledgment statement can be found on the LONI site: https://adni.loni.usc.edu/wp-content/uploads/how_to_apply/ADNI_Data_Use_Agreement.pdf.

### FASe_Families (sa000004) data:

This work was supported by grants from the National Institutes of Health (R01AG044546, P01AG003991, RF1AG053303, R01AG058501, U01AG058922, RF1AG058501 and R01AG057777). The recruitment and clinical characterization of research participants at Washington University were supported by NIH P50 AG05681, P01 AG03991, and P01 AG026276. This work was supported by access to equipment made possible by the Hope Center for Neurological Disorders, and the Departments of Neurology and Psychiatry at Washington University School of Medicine.

We thank the contributors who collected samples used in this study, as well as patients and their families, whose help and participation made this work possible. This work was supported by access to equipment made possible by the Hope Center for Neurological Disorders, and the Departments of Neurology and Psychiatry at Washington University School of Medicine

### KnightADRC (sa000008) data:

This work was supported by grants from the National Institutes of Health (R01AG044546, P01AG003991, RF1AG053303, R01AG058501, U01AG058922, RF1AG058501 and R01AG057777). The recruitment and clinical characterization of research participants at Washington University were supported by NIH P50 AG05681, P01 AG03991, and P01 AG026276. This work was supported by access to equipment made possible by the Hope Center for Neurological Disorders, and the Departments of Neurology and Psychiatry at Washington University School of Medicine.

We thank the contributors who collected samples used in this study, as well as patients and their families, whose help and participation made this work possible. This work was supported by access to equipment made possible by the Hope Center for Neurological Disorders, and the Departments of Neurology and Psychiatry at Washington University School of Medicine.

### AMP-AD (sa000011) data:

Mayo RNAseq Study- Study data were provided by the following sources: The Mayo Clinic Alzheimer's Disease Genetic Studies, led by Dr. Nilufer Ertekin-Taner and Dr. Steven G. Younkin, Mayo Clinic, Jacksonville, FL using samples from the Mayo Clinic Study of Aging, the Mayo Clinic Alzheimer's Disease Research Center, and the Mayo Clinic Brain Bank. Data collection was supported through funding by NIA grants P50 AG016574, R01 AG032990, U01 AG046139, R01 AG018023, U01 AG006576, U01 AG006786, R01 AG025711, R01 AG017216, R01 AG003949, NINDS grant R01 NS080820, CurePSP Foundation, and support from Mayo Foundation. Study data includes samples collected through the Sun Health Research Institute Brain and Body Donation Program of Sun City, Arizona. The Brain and Body Donation Program is supported by the National Institute of Neurological Disorders and Stroke (U24 NS072026 National Brain and Tissue Resource for Parkinson's Disease and Related Disorders), the National Institute on Aging (P30 AG19610 Arizona Alzheimer's Disease Core Center), the Arizona Department of Health Services (contract 211002, Arizona Alzheimer's Research Center), the Arizona Biomedical Research Commission (contracts 4001, 0011, 05-901 and 1001 to the Arizona Parkinson's Disease Consortium) and the Michael J. Fox Foundation for Parkinson's Research

ROSMAP- We are grateful to the participants in the Religious Order Study, the Memory and Aging Project. This work is supported by the US National Institutes of Health [U01 AG046152, R01 AG043617, R01 AG042210, R01 AG036042, R01 AG036836, R01 AG032990, R01 AG18023, RC2 AG036547, P50 AG016574, U01 ES017155, KL2 RR024151, K25 AG041906-01, R01 AG30146, P30 AG10161, R01 AG17917, R01 AG15819, K08 AG034290, P30 AG10161 and R01 AG11101.

Mount Sinai Brain Bank (MSBB)- This work was supported by the grants R01AG046170, RF1AG054014, RF1AG057440 and R01AG057907 from the NIH/National Institute on Aging (NIA). R01AG046170 is a component of the AMP-AD Target Discovery and Preclinical Validation Project. Brain tissue collection and characterization was supported by NIH HHSN271201300031C.

### UPitt Kamboh (sa000012) data:

This study was supported by the National Institute on Aging (NIA) grants AG030653, AG041718, AG064877 and P30-AG066468.

### NACC Genentech (sa000013) data:

We would like to thank study participants, their families, and the sample collectors for their invaluable contributions. This research was supported in part by the National Institute on Aging grant U01AG049508 (PI Alison M. Goate). This research was supported in part by Genentech, Inc. (PI Alison M. Goate, Robert R. Graham).

The NACC database is funded by NIA/NIH Grant U01 AG016976. NACC data are contributed by these NIA-funded ADCs: P30 AG013846 (PI Neil Kowall, MD), P50 AG008702 (PI Scott Small, MD), P50 AG025688 (PI Allan Levey, MD, PhD), P30 AG010133 (PI Andrew Saykin, PsyD), P50 AG005146 (PI Marilyn Albert, PhD), P50 AG005134 (PI Bradley Hyman, MD, PhD), P50 AG016574 (PI Ronald Petersen, MD, PhD), P30 AG013854 (PI M. Marsel Mesulam, MD), P30 AG008017 (PI Jeffrey Kaye, MD), P30 AG010161 (PI David Bennett, MD), P30 AG010129 (PI Charles DeCarli, MD), P50 AG016573 (PI Frank LaFerla, PhD), P50 AG005131 (PI Douglas Galasko, MD), P30 AG028383 (PI Linda Van Eldik, PhD), P30 AG010124 (PI John Trojanowski, MD, PhD), P50 AG005142 (PI Helena Chui, MD), P30 AG012300 (PI Roger Rosenberg, MD), P50 AG005136 (PI Thomas Grabowski, MD), P50 AG005681 (PI John Morris, MD), P30 AG028377 (Kathleen Welsh-Bohmer, PhD), and P50 AG008671 (PI Henry Paulson, MD, PhD).

Samples from the National Cell Repository for Alzheimer’s Disease (NCRAD), which receives government support under a cooperative agreement grant (U24 AG21886) awarded by the National Institute on Aging (NIA), were used in this study. We thank contributors who collected samples used in this study, as well as patients and their families, whose help and participation made this work possible.

The Alzheimer's Disease Genetics Consortium supported the collection of samples used in this study through National Institute on Aging (NIA) grants U01AG032984 and RC2AG036528.

### CacheCounty (sa000014) data:

We acknowledge the generous contributions of the Cache County Memory Study participants. Sequencing for this study was funded by RF1AG054052 (PI: John S.K. Kauwe).
